# Low input fast-track (LIFT): an approach for fast introgression and stacking of (*R*-)genes into advanced apple selections

**DOI:** 10.1007/s00425-025-04780-4

**Published:** 2025-09-02

**Authors:** Simone Bühlmann-Schütz, Marius Hodel, Nicholas P. Howard, Luzia Lussi, Andrea Patocchi

**Affiliations:** 1 Agroscope, Research Division Plant Breeding, Müller-Thurgau-Strasse 29, 8820 Waedenswil, Switzerland; 2 FRESH-FORWARD, Wielseweg 38a, 4024 BK Eck en Weil, Netherlands

**Keywords:** Fire blight, *Malus*, Marker-assisted selection (MAS), Resistance breeding, Speed breeding

## Abstract

Using the “LIFT” method can halve generation time to two years, thereby reducing the breeding cycle by 50%, and accelerate the development of disease-resistant apple cultivars for sustainable production.

Good sources of resistance to pests and pathogens are often found in wild relatives or ornamental apples, which are mainly small-fruited and poor-tasting. Introgressing these resistance genes via classical breeding into new apple cultivars with good tree and fruit qualities requires a series of four to five pseudo-backcrosses, which takes at least 25 years. This study aimed to develop a low-input protocol to shorten the time between generations and thereby substantially reduce the timeline for the development of cultivars with introgressed resistance. The “LIFT” method combined forced growth of seedlings carrying the fire blight resistance genes from ‘Evereste’ or from *Malus* × *robusta* 5 in a standard greenhouse with artificial vernalization periods in a cold room. This allowed flower induction in about 20% of the seedlings as early as after the second vernalization period, i.e., about two years after sowing. The fire blight resistance, the increase in single fruit weight, and the reduction of the unadapted part of the genome derived from ‘Evereste’ or *Malus* × *robusta* 5 were monitored across generations. No erosion of the resistance was observed, but there was an increase in single fruit weight and a progressive reduction of the unadapted genome. Therefore, the developed protocol proved to be efficient and reliable and can be applied with or without molecular markers to introduce or combine traits from wild apple relative into advanced pre-breeding selections. These pre-breeding selections are urgently needed to develop new apple cultivars with broad disease resistance, ensuring long-term sustainable apple fruit production.

**Supplementary Information:**

The online version contains supplementary material available at 10.1007/s00425-025-04780-4.

## Introduction

Apples are the third most cultivated fruit crop worldwide (FAO 2023). However, their production requires a significant use of plant protection products, as the most commonly grown cultivars are susceptible to many diseases and pests, such as the fungal diseases apple scab (*Venturia inaequalis* (Cke.) Wint.) and powdery mildew (*Podosphaera leucotricha* (Ellis and Everh.) E. S. Salmon), or the bacterial disease fire blight caused by *Erwinia amylovora* (Burrill) (Winslow et al. 1920). Growing cultivars that are resistant or less susceptible to these and other diseases would allow for a substantial reduction in the use of plant protection products. Therefore, disease resistance has been a major topic in many breeding programs worldwide for decades.

The first resistance to apple scab was discovered in the United States at the beginning of the twentieth century with the discovery of complete field immunity in some wild-type selections of *Malus* species (Hough 1944). However, it took more than four decades from the initial first cross in 1914 (Crandall 1926) to the final cross in 1958, which led to the launch of the first intentionally bred apple scab-resistant cultivar “Prima” in 1967 by the cooperative breeding program Purdue, Rutgers, Illinois (PRI) (Papp et al. 2020). The apple scab resistance gene (*R*-gene) carried by ‘Prima’, *Rvi6* alias *Vf* from *Malus floribunda* 821 (Crandall 1926), is still the most widely used *R*-gene in apple breeding today (Brown and Maloney 2013). However, in contrast to susceptible cultivars, the market share of apple scab-resistant cultivars remains very low (Nuijten et al. 2018). In addition, the breakdown of the *Rvi6 R*-gene has been registered since the mid-1980s (Parisi et al. 1993). Fortunately, alternative good sources of resistance to apple scab (reviewed in Bowen et al. 2011; Broggini et al. 2011; Khajuria et al. 2018) and other diseases, such as powdery mildew (Strickland et al. 2021) and fire blight (reviewed in Emeriewen et al. 2019), have been identified over the years. However, most of these *R*-genes are found in wild relatives or ornamental apples, which mainly have small fruits and poor taste. The use of such resistance sources for breeding new cultivars with good fruit quality requires about four to five pseudo-backcrosses (pBCs) to eliminate undesirable traits. However, the juvenility period in modern breeding programs is typically four to five years. This means it would take up to 25 years to introgress a new *R*-gene from a wild relative. Therefore, breeders are looking for approaches to shorten the juvenile phase of apple trees.

In the last decade, several transgenic approaches to reduce the juvenile phase in apple have been developed. Flachowsky et al. (2007) and Kotoda et al. (2010) overexpressed the FRUITFULL homolog *BpMADS4* of silver birch (*Betula pendula* Roth.) and the apple FLOWERING LOCUS T (FT), respectively. Whereas Kotoda et al. (2006) used RNA interference to silence the *MdTFL1* gene, Charrier et al. (2019) used CRISPR/Cas9 to knock out the same gene. All of these approaches have reduced the juvenile phase of apples in general to less than one year. Concluding the work started by Le Roux et al. (2012), Schlathölter et al. (2018) used the “early flowering” line T1190, which overexpresses the *BpMADS4* gene (Flachowsky et al. 2007), to show that it was possible to perform five pBCs within seven years and select highly fire blight-resistant null segregant lines in the last cross. USDA (U.S. Department of Agriculture) regulators consider such lines to be outside their regulatory authority, as long as these individuals have been phenotypically tested and molecularly proven to be free of transgenes or pieces of transgenes (USDA 2011, 2014; McGarry et al. 2017). The definition of the legal status of the final products in the European Union is still pending, and, therefore, European breeders are looking for agrotechnical approaches. To date, the most promising approach was proposed by Volz et al. (2009). The highly sophisticated method involved growing seedlings on their own roots in a growth chamber under optimal conditions, i.e. high temperatures (26–30 °C), high relative humidity (85%), high irradiance (> 900 µmol m^2^ s^−1^), long photoperiod (18 h per day), high red:far-red (R:FR) ratio, elevated CO_2_ concentration (approx. 1,800 ppm CO_2_), non-limiting fertigation, and the application of Prohexadione-Ca (also known as BX-112). With this approach, Volz et al. (2009) succeeded in reducing the time to flowering to about 18 months from seed sowing. This method represents a significant advancement in accelerating the breeding cycle for apples.

Inspired by Volz et al.’s (2009) study, the present work aimed to develop a low-input approach, which we called the “low input fast-track” (LIFT) method, using a normal greenhouse to reduce costs and enable the inclusion of a relatively large number of plants. The method developed pursues the same goal, namely to reduce the time between generations to enable an accelerated generation cycle for introgression breeding. While developing the method, advanced breeding selections carrying the fire-blight *R*-genes of ‘Evereste’ (*Fb_E*, Durel et al. 2009) and *Malus* × *robusta* 5 (*FB_MR5*, Peil et al. 2007) were produced. Fire blight resistance and single fruit weight were phenotypically monitored across generations. Finally, we tracked the unadapted exotic haplotypes of the resistance donor parents present in several descendant seedlings from the fourth or fifth pBC generation and compared our outcome with the lines developed using the “early flowering” approach by Schlathölter et al. (2018).

## Materials and methods

### Plant material

The development of advanced selections using the “LIFT” method, carrying the fire blight *R*-genes *Fb_E* from ‘Evereste’ (Durel et al. 2009) and *FB_MR5 from Malus* × *robusta* 5 (MR5) (Peil et al. 2007) was initiated using ‘Evereste’ and three F1 selections DA02. 2.7, DA02. 2.40, and DA02. 1.27 (all derived from an ‘Idared’ × MR5 cross at Julius Kühn-Institut (JKI), Dresden, Germany) as resistance donor parents. Crosses with these selections were made under field conditions according to Baumgartner et al. (2015) at the Federal Research Station Agroscope in Waedenswil, Switzerland, in 2008 and 2009. A list of all cross combinations carried out over the years, including the used modern parental cultivars is provided in Suppl. Table S1. Pollen of the three *FB_MR5* F1 selections was kindly provided by Dr. Andreas Peil (JKI, Dresden, Germany). The plant material established using the “early flowering” line T1190 (Flachowsky et al. 2011) was described by Schlathölter et al. (2018). This material is used to compare the two methods: “early flowering” versus “LIFT.”

### Low-input fast-track (LIFT)

**Fig. 1:**
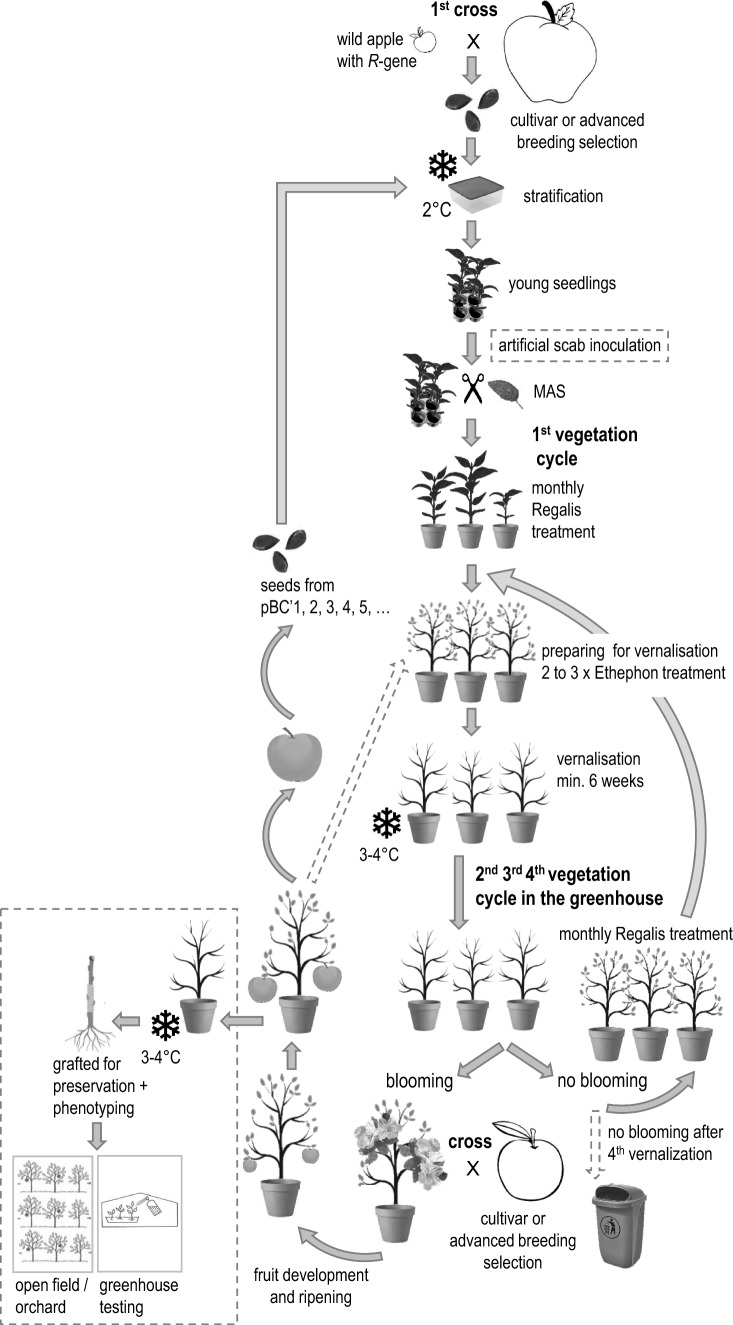
Schematic representation of the low input fast-track (LIFT) method. The initial cross (1st cross) between the resistance gene donor and a modern cultivar, from which the seeds of the F1 generation emerged, was followed by seed stratification, sowing, and growing of the seedlings in the greenhouse, the application of an artificial scab inoculation on seedlings from specific crosses in the greenhouse, and MAS. The selected seedlings were potted and introduced into the first vegetative cycle in the greenhouse, followed by a vernalization period of at least 6 weeks. After the cold treatment, the seedlings were brought back into the greenhouse for the next vegetative growth period, and the cycle was repeated until flowering. Once a seedling flowered, the anthers were collected, and the flowers were pollinated to obtain the next generation. Ripe fruits were harvested, the seeds were extracted, and the cycle was restarted with seed stratification. Seedlings that did not flower after the fourth vernalization period were discarded. Seedlings that flowered in the greenhouse were placed in a cold store to obtain budwood for grafting and multiplication for further agronomic testing under field conditions or artificial disease screenings in the greenhouse

The latest version of the “LIFT” protocol is described below (Fig. 1). A few parameters of the method have been adapted and optimized over the years (2008–2024) (e.g., duration of vernalization and concentration of Prohexadione-Ca treatment). Seeds were extracted from fruits harvested at maturity from each cross and stratified in humid sand (2 °C, approx. 95% humidity) for about 2.5 months. The seeds were then sown in nursery trays (Quick Pot QP 35 T or QP 24 T/13 from HerkuPlast-Kubern GmbH (Ering, Germany) filled with a “B Cutting” substrate (Floragard GmbH, Oldenburg, Germany). After germination, the seedlings were cultivated under greenhouse conditions (approx. 15–17 °C). At the four-leaf stage, a leaf sample was collected from each seedling for marker-assisted selection (MAS) (see below), or the seedlings of populations expected to segregate for one or more *R*-genes against apple scab (*Venturia inaequalis*) were inoculated with scab according to Bühlmann-Schütz et al. (2023) before MAS.

The most vigorous seedlings with the desired (combination of) *R*-gene(s) were introduced into the “LIFT” system. “LIFT” seedlings were transferred to 4.5 L pots filled with “FLORADUR Special Gardener’s Soil Lawn” substrate (Floragard GmbH) in a regular greenhouse under warm, long-day conditions (approx. 20° C, approx. 60% RH, approx. 14 h of illumination with a 600-Watt high-pressure sodium lamp) and non-limiting fertilization by drip irrigation (1.5% YaraTera Kristalon Rot Calcium, YARA GmbH and Co. KG, Dülmen, Germany). A sulfur vaporizer (Hotbox Sulfume from Hotbox International Limited, East Yorkshire, UK) was switched on for 1 h each night to control powdery mildew. The biocontrol product Solbac (Andermatt Biocontrol AG, Grossdietwil, Switzerland) was applied twice after potting against the larvae of the dark-winged fungus gnats (Sciaridae). If needed, other insecticides were used to control various pests, e.g., green apple aphids, woolly apple aphids, whiteflies, or spider mites. Regalis® Plus (Stähler Suisse SA, Zofingen, Switzerland), containing 0.08% Prohexadione-Ca, a gibberellins biosynthesis inhibitor, was applied monthly to reduce internode length without affecting node production. After 5–6 months of active plant growth in the greenhouse, leaf senescence and leaf fall were induced by weekly application of the growth and ripening regulator Ethephon, containing 0.1% 2-chloroethylphosphoric acid (SINTAGRO M. Eggen, Langenthal, Switzerland), for two to three consecutive weeks. The vernalization of the seedlings was carried out in a normal cold store at 3–4 °C for an average of 11 weeks (min. 6 weeks). In the few cases where all the seedlings could not fit in the cold store, the seedlings in excess were vernalized by exposing them to the winter conditions of the specific year. After the vernalization period, if all the seedlings could not be placed back in the greenhouse, the seedlings in excess were placed in plastic tunnels during the summer. Since these trees did not meet the standard protocol, they were excluded from the analysis of flower induction. After vernalization, the seedlings were returned to the greenhouse under the same conditions as previously described. On average, the seedlings were kept under vegetative conditions for around 5–6 months between the vernalization periods. Very unhealthy seedlings and seedlings with reduced growth were removed in each cycle before vernalization or after budbreak back in the greenhouse. Once the seedlings flowered, they were emasculated for pollen collection. These flowers were subsequently pollinated with pollen from modern cultivars or advanced breeding selections with superior fruit quality traits and, when possible, low fire blight susceptibility and *R*-genes against apple scab and mildew or the fire blight QTL *FB_F7*. The pollen from these modern cultivars or advanced breeding selections was collected annually in the field during the regular flowering period in spring, air-dried, and stored at −80 °C until use. The pollen collected from the seedlings in the greenhouse was also air-dried and then stored at −80 °C until it was used to pollinate the flowers of modern cultivars or advanced breeding selections cultivated in the field. Progenies carrying the *Fb_E* or *FB_MR5 R*-genes that produced flowers were used as parents for the next introgression cycle. Depending on the seedling, fruits were harvested approx. 4–6 months after pollination in the greenhouse. Seeds from harvested fruits were extracted, dried, and either stratified as previously described or stored at −24 °C until stratification.

### Agronomic testing and preservation in the field

Seedlings that flowered in the greenhouse were grafted onto the rootstock M.27 with a ‘Schneiderapfel’ interstem to preserve them and further evaluate their fruit quality under field conditions or directly onto M.9vf T337 to verify their fire blight resistance level in an artificial fire blight inoculation test in the greenhouse (see below). Due to the limited space available in the “LIFT” system (capacity for approx. 200 plants), some descendants were grafted directly onto the rootstock M.27 interstem ‘Schneiderapfel’ or directly onto M.9vf T337 after MAS and planted in the field after 1 year in the nursery. All trees in the field were managed in accordance with the guidelines for integrated production. In the first year after planting, all flowers were removed by hand to promote vegetative growth of the trees. After that, manual thinning was carried out every year shortly after the natural fruit shedding (also known as ‘June drop’).

### Marker-assisted selection—Foreground selection of seedlings

The molecular markers listed in Table 1 (primer sequences are reported in Suppl. Table S2) were used to identify the seedlings carrying the fire blight *R*-genes *FB_E or FB_MR5*, as well as the apple scab *R*-genes *Rvi2, Rvi4, Rvi6*, the powdery mildew *R*-genes *Pl1, Pl2*, or the fire blight resistance QTL *FB_F7*, if it was assumed that these genes would segregate in the progeny. MAS was performed according to Bühlmann-Schütz et al. (2023). By 2019, one frozen leaf sample per seedling was prepared and shipped to Ecogenics GmbH (Balgach, Switzerland) for genotyping with simple sequence repeat (SSR) and sequence-characterized amplified region (SCAR) markers using multiplex PCR assays with fluorescent-labeled primers. From 2020 onward, one leaf disk per seedling was collected, dried, and sent to LGC Genomics Ltd. (Teddington, UK) for genotyping with single-nucleotide polymorphism (SNP) markers using the KASP™ PCR assays.

**Table 1:** Molecular markers used for MAS

***R*-gene or QTL**	**LG**^a^	**Marker name**	**Marker type**	**Allele in coupling** ^b^	**Reference**
*Fb_E*	12	ChFbE06 FBsnFBE-1_Y230 FBsnFBE-2_Y495	SSR SNP SNP	260 (243/243) C C	Parravicini et al. (2011) Jänsch et al. (2015) Jänsch et al. (2015)
*FB_MR5*	3	FEM47 FEM19 FB-MR5_SNP_M106 FB-MR5_SNP_R209	SSR SSR SNP SNP	218 (202/225) 157 (145/165) A A	Fahrentrapp et al. (2013) Fahrentrapp et al. (2013) Jänsch et al. (2015) Jänsch et al. (2015)
*FB_F7*	7	AE10-375 GE-8019 SNP_FB_0716011 SNP_FB_0716013	SCAR SCAR SNP SNP	375 (0) 397 (0) A C	Khan et al. (2007) Khan et al. (2007) van de Weg et al. (2018) van de Weg et al. (2018)
*Rvi2*	2	CH05e03	SSR	173 (179/190)	Bus et al. (2005)
		OPL19	SCAR	438 (0)	Bus et al. (2005)
		FbsnRvi2-1_M417	SNP	A	Jänsch et al. (2015)
		FBsnRvi2-2_M341	SNP	C	Jänsch et al. (2015)
		FBsnRvi2-3_M58	SNP	T	Jänsch et al. (2015)
		FBsnRvi2-4_R489	SNP	A	Jänsch et al. (2015)
		FBsnRvi2-5_M366	SNP	A	Jänsch et al. (2015)
		FBsnRvi2-6_1_M95	SNP	A	Jänsch et al. (2015)
		FBsnRvi2-6_2_M133	SNP	C	Jänsch et al. (2015)
		FBsnRvi2-7_Y292	SNP	C	Jänsch et al. (2015)
		FBsnRvi2-8_R243	SNP	G	Jänsch et al. (2015)
*Rvi4*	2	CH02C02a	SSR	182 (148/184)	Bus et al. (2005)
		Hi22d06	SSR	132 (132/138)	Silfverberg-Dilworth et al. (2006)
		FBsnRvi4.1_K146	SNP	T	Jänsch et al. (2015)
		TNL1_Rvi4_R131	SNP	G	Jänsch et al. (2015)
*Rvi6*	1	CH-Vf1	SSR	164 (146/146)	Vinatzer et al. (2004)
		M8S_Rvi6_Y124	SNP	T	Jänsch et al. (2015)
		M18_Rvi6_Y32	SNP	T	Jänsch et al. (2015)
*Pl1*	12	AT20 SNP_12_30034791 SNP_12_30781908	SCAR SNP SNP	458 (0) A A	Dunemann et al. (2007) García-Gómez et al. (2023) García-Gómez et al. (2023)
*Pl2*	11	CH04H02 FBsnPl2-1_Y245 FBsnPl2-1_R531	SSR SNP SNP	186 (184/200) C G	Rikkerink et al. (2007) Jänsch et al. (2015) Jänsch et al. (2015)

Simple-sequence repeat (SSR), sequence characterized amplified region (SCAR), and single-nucleotide polymorphism (SNP) markers associated with a specific resistance gene (*R*-gene) or quantitative trait locus (QTL) used for MAS (adapted from Bühlmann-Schütz et al. 2023) (primer sequences in Suppl. Table S2)

^a^ Linkage group

^b^ The allele sizes of ‘Gala Galaxy’ are given in brackets for reference

### Fire blight resistance assessment – artificial shoot inoculation

Seedlings that flowered in the greenhouse, from which pollen was collected for pollination in the field, or from which fruits were harvested, were grafted on rootstock M.9vf T337. The grafts were potted in rose pots (Stuewe and Sons, Inc., Tangent, OR, USA; 35.5 cm pot height, 7 cm diameter, 12 replicate trees per individual) for the artificial fire blight shoot inoculation test according to Bühlmann-Schütz et al. (2024). ‘Gala Galaxy’ and ‘Enterprise’ were included in each trial as susceptible and resistant controls, respectively. To compare the susceptibility level of the tested individuals between trials and years, the percent lesion length (lesion length divided by total shoot length) was calculated in relation to the percent lesion length of the susceptible control ‘Gala Galaxy’ at 21 days after inoculation.

### Assessment of single fruit weight

The fruits of all the trees planted in the field were harvested every year from the second leaf. The fruits were harvested per tree when at least 50% of the fruits were physiologically fully mature, as determined by expert knowledge. Single fruit weight was assessed after harvest using FruitPhenoBox (Kirchgessner et al. 2024) or manual weighing and counting. This trait was analyzed for up to 3 years (number of analyzed individuals per year: 122, 57, and 12, respectively), and the collected data were aggregated per generation to compare the trait across generations.

### SNP genotyping and pedigree reconstruction

All breeding selections and crossing parents were genotyped on the Illumina Infinium 20 K SNP array (Bianco et al. 2014). SNP data were processed and curated as described by Vanderzande et al. (2019). A set of 10,321 highly curated SNPs from the 20 K array that were used in several previous publications (e.g., Howard et al. 2021a; Volk et al. 2022; Larsen et al. 2025) were used for this study Suppl. Table S3 and S4. These SNP data were combined with data from an ongoing collaborative large-scale apple pedigree reconstruction project (Howard et al. 2018) for pedigree reconstruction and to improve data curation. Mendelian error rates were used to identify parent–offspring relationships, as described by Vanderzande et al. (2019). A comparison of shared haplotype information was used to identify more distant relationships, as described by Howard et al. (2021a). The unadapted exotic material used in this study had a tendency toward having null alleles, which obscured the identification or confirmation of true pedigree relationships. To address this, cluster plots of SNPs that had Mendelian errors in recorded pedigrees were inspected in GenomeStudio v2.0 (Illumina Inc.) to ensure that the errors were due to null alleles and not to incorrect pedigree records. SNP calls were adjusted accordingly to address these Mendelian errors and to enable haplotype tracing. The resulting pedigree and curated SNP data were used for phasing of the SNP data in FlexQTL (Bink et al. 2014). The genetic map used was an edited version of the iGLmap (Di Pierro et al. 2016) described by Howard et al. (2021b).

### Introgression tracking across generations and comparison with individuals produced using “early flowering” or “LIFT”

Haplotypes over the *Fb_E* and *FB_MR5 R*-genes, and the unadapted portions of each introgressed selection were traced through each generation. The position of *FB_MR5* was determined by BLASTing the FB-MR5_NZsnEH034548_K35, FB-MR5_rp16k15_M106, FB-MR5_FBsnFBMr5-1_Y127, and FB-MR5_FBsnFBMr5-1_R209 SNPs from Jänsch et al. (2015) (Suppl. Table S5) against the GDDH13v1.1 whole genome sequence (Daccord et al. 2017) and locating flanking SNPs from the curated set from the 20 K SNP array (Suppl. Table S6). Similarly, *Fb_E* was positioned using SNPs FB-E_FBsnFBE-1_Y230, FB-E_FBsnFBE-2_Y192, FB-E_FBsnFBE-2_Y495, and FBsnFBE-2_Y551 from Jänsch et al. (2015) (Suppl. Table S5). The correct homologs containing these *R*-genes were determined by comparing phased data between the resistance donors MR5 and ‘Evereste’, respectively, and their first-generation descendants previously recorded as having the *R*-genes via SSR analysis and determining which haplotypes were held in common over the regions where the *R*-genes were positioned.

Unadapted portions of the genomes of selections were defined as those that did not originate from *M. domestica* cultivars or, in the case of ‘Evereste’, portions not originating from its identified parent. This distinction was made because one parent of ‘Evereste’ was identified as an advanced breeding selection that had some introgressed haplotypes from *M. floribunda* 821. The unadapted portions in MR5 and ‘Evereste’ were assumed to have come from tiny-fruited wild *Malus* species. Unadapted *Malus* haplotypes were traced by first defining them in MR5 and ‘Evereste’ by subtracting the domestic haplotypes. The remaining haplotypes were then compared to the haplotypes of the descendants of MR5 and ‘Evereste’ to determine which were present in the descendants. A shared haplotype was considered as starting and stopping at shared SNPs either at the ends of chromosomes or at shared SNPs adjacent to SNPs that were not shared. The haplotypes shared between the resistance donors and their descendants had to be greater than 15 SNPs and not to be present in the non-admixed parent (or shared by both, but the extended region on either side had to be present in the resistant donor and not in the non-admixed parent). The unadapted portion of the genome of each selection was recorded in centimorgans (cM). The Python script HapShared (Howard et al. 2021a) was used to perform these comparisons, but they were also determined manually in Excel to ensure accuracy due to the high frequency of null alleles despite the curation process.

### Statistical data analysis

One-way ANOVA tests were performed to assess the effect of generation on single fruit weight and fire blight resistance. Letters indicating significant groups (*P* < 0.05) were assigned after a post-hoc Tukey test. All statistical analyses and data preparation were performed in R (4.4.1; R Core Team 2024) using the following packages: *RSQLite* (v2.3.1; Müller et al. 2022), *openxlsx* (v4.2.5.2; Schauberger and Walker 2021) and the *tidyverse* (Wickham et al. 2019). Packages *dplyr* (v1.1.2), *tidyr* (v1.3.0), and *magrittr* (v2.0.3) were used to load and parse the data. Packages *ggplot2* (v3.4.3; Wickham et al. 2022), *patchwork (v1.1.3;* Pederson 2023), and *multcompView* (v0.1–9; Graves et al. 2019) were utilized to visualize the results.

## Results

### Comparison of plant material derived from crosses in “LIFT” with crosses from the field

Seedlings introduced to “LIFT” had two different origins. They either descended from “LIFT” mother plants grown in the greenhouse and pollinated with pollen from the field, or from trees in the field pollinated with pollen from “LIFT”. In both introgression series (*Fb_E* and *FB_MR5*), over all six generations, a higher number of crosses were made in “LIFT” than in the field (on average, 19.2 vs. 4 crosses, Tables 2 and 3). However, only 27.4% of all the flowers were pollinated in “LIFT”. Consequently, more fruits and seeds were produced in the field. Across all generations carrying *Fb_E*, the average number of harvested single fruits per pollinated flower was essentially the same for crosses originating from the field or the “LIFT”, with the value from the “LIFT” (0.18) being slightly higher than from the field (0.14). For *FB_MR5*, this ratio was higher for the crosses made in the field (0.2 vs. 0.12). Regarding the average number of seeds per fruit across generations, for both fire blight *R*-genes, the fruits produced in “LIFT” contained about 0.6 more seeds per fruit than those produced in the field. However, 63.3% and 82.4% of the seeds generated with *Fb_E* and *FB_MR5*, respectively, originated from large crosses performed in the field.

**Table 2:** Summary of crosses, pollinated flowers, number of seedlings produced, and seedlings carrying the fire blight resistance gene *Fb_E* from crosses made in the field or in “LIFT” (location) across the introgression series

	Location	F1	%*	pBC1	%*	pBC2	%*	pBC3	%*	pBC4	%*	pBC5	%*	Total	%*	Average across generations	%*
Number of crosses	Field	2		7		5		2		4		0		20		4.0	
	LIFT	0		13		14		34		32		3		96		19.2	
	Total	**2**		**20**		**19**		**36**		**36**		**3**		**116**			
Number of pollinated flowers	Field	1288		992		1329		134		1068		0		4811	72.6	962.2	
	LIFT	0		328		247		457		707		74		1813	27.4	362.6	
	Total	**1288**		**1320**		**1576**		**591**		**1775**		**74**		**6624**			
Average number of pollinated flowers per cross	Field	644		142		266		67		267		NA				277.1	
	LIFT	NA		25		18		13		22		25				20.6	
Number of harvested single fruits	Field	227		77		98		21		231		0		654	67.2	130.8	
	LIFT	0		119		41		88		64		7		319	32.8	63.8	
	Total	**227**		**196**		**139**		**109**		**295**		**7**		**973**			
Average number of harvested single fruits per pollinated flower	Field	0.18		0.08		0.07		0.16		0.22		NA				0.14	
	LIFT	NA		0.36		0.17		0.19		0.09		0.09				0.18	
Number of seeds	Field	977		279		574		119		1284		0		3233	64.2	646.6	
	LIFT	0		507		218		572		472		31		1800	35.8	360.0	
	Total	**977**		**786**		**792**		**691**		**1756**		**31**		**5033**			
Average number of seeds per fruit	Field	4		4		6		6		6		NA				5	
	LIFT	0		4		5		7		7		4				5.6	
Number of sown seeds	Field	977		279		518		119		731		0		2624	63.3	524.8	
	LIFT	0		287		157		572		472		31		1519	36.7	303.8	
	Total	**977**		**566**		**675**		**691**		**1203**		**31**		**4143**			
Number of seedlings eliminated after scab screening	Field	3		0		72		10		115		0		200		40.0	7.6
	LIFT	0		0		0		0		11		0		11		2.2	0.7
	Total	**3**		**0**		**72**		**10**		**126**		**0**		**211**			
Number of seeds that failed to germinate, or seedlings that died or were discarded for various reasons before MAS	Field	395	40.4	31	11.1	272	52.5	92	77.3	242	33.1	0	NA	1032		206.4	39.3
	LIFT	0	NA	125	43.6	84	53.5	220	38.5	97	20.6	4	13	530		106.0	34.9
	Total	**395**		**156**		**356**		**312**		**339**		**4**		**1562**			
Number of tested seedlings (MAS)	Field	579		248		174		17		374		0		1392	58.7	278.4	53.0
	LIFT	0		162		73		352		364		27		978	41.3	195.6	64.4
	Total	**579**		**410**		**247**		**369**		**738**		**27**		**2370**			
Number of seedlings with *R*-gene	Field	279	48.2	92	37.1	105	60.3	6	35.3	167	44.7	0	0	649		129.8	45.1
	LIFT	0	0.0	49	30.2	39	53.4	181	51.4	194	53.3	14	52	477		95.4	48.0
	Total	**279**		**141**		**144**		**187**		**361**		**14**		**1126**			
Number of seedlings selected for LIFT	Field	57		81		21		0		75		0		234		46.8	
	LIFT	0		40		30		129		92		10		301		60.2	
	Total	**57**		**121**		**51**		**129**		**167**		**10**		**535**			
Number of parents in next LIFT generation	Field	16		6		9		0		2		0		33		6.6	
	LIFT	0		2		11		13		0		0		26		5.2	
	Total	**16**		**8**		**20**		**13**		**2**		**0**		**59**			

^*^ % have been calculated only for selected cells; Bold: Totals

**Table 3:** Summary of crosses, pollinated flowers, number of seedlings produced, and seedlings carrying the fire blight resistance gene *FB_MR5* from crosses made in the field or in “LIFT” (location) across the introgression series

	Location	pBC1	%*	pBC2	%*	pBC3	%*	pBC4	%*	pBC5	%*	pBC6	%*	Total	%*	Average across generations	%*
Number of crosses	Field	3		10		6		5		3		2		29		4.8	
	LIFT	0		1		51		33		3		10		98		19.6	
	Total	**3**		**11**		**57**		**38**		**6**		**12**		**127**			
Number of pollinated flowers	Field	1129		1452		1006		1110		1107		373		6177	80.4	1029.5	
	LIFT	0		15		545		715		43		188		1506	19.6	301.2	
	Total	**1129**		**1467**		**1551**		**1825**		**1150**		**561**		**7683**			
Average number of pollinated flowers per cross	Field	376		145		168		222		369		187				213.0	
	LIFT	NA		15		11		22		14		19				15.4	
Number of harvested single fruits	Field	242		288		182		118		333		55		1218	86.9	203.0	
	LIFT	0		10		84		85		0		5		184	13.1	36.8	
	Total	**242**		**298**		**266**		**203**		**333**		**60**		**1402**			
Average number of harvested single fruits per pollinated flower	Field	0.21		0.20		0.18		0.11		0.30		0.15				0.20	
	LIFT	NA		0.67		0.15		0.12		0.00		0.03				0.12	
Number of seeds	Field	1100		1809		960		602		800		197		5468	83.7	911.3	
	LIFT	0		59		407		559		0		36		1061	16.3	212.2	
	Total	**1100**		**1868**		**1367**		**1161**		**800**		**233**		**6529**			
Average number of seeds per fruit	Field	4.5		6.3		5.3		5.1		2.4		3.6				4.5	
	LIFT	NA		5.9		4.8		6.6		0.0		7.2				4.9	
Number of sown seeds	Field	1098		1491		960		602		800		13		4964	82.4	827.3	
	LIFT	0		59		407		559		0		36		1061	17.6	212.2	
	Total	**1098**		**1550**		**1367**		**1161**		**800**		**49**		**6025**			
Number of seedlings eliminated after scab screening	Field	353		183		124		144		301		0		1105		184.2	22.3
	LIFT	0		0		0		0		0		0		0		0.0	0.0
	Total	**353**		**183**		**124**		**144**		**301**		**0**		**1105**			
Number of seeds that failed to germinate, or seedlings that died or were discarded for various reasons before MAS	Field	134	12.2	626	42.0	228	23.8	127	21.1	207	25.9	2		1324		404.8	26.7
	LIFT	0		38	64.4	123	30.2	81	14.5	0	NA	12		254		50.8	23.9
	Total	**487**		**847**		**475**		**352**		**508**		**14**		**2683**			
Number of tested seedlings (MAS)	Field	611		682		608		331		292		11		2535	75.9	422.5	51.1
	LIFT	0		21		284		478		0		24		807	24.1	161.4	76.1
	Total	**611**		**703**		**892**		**809**		**292**		**35**		**3342**			
Number of seedlings with *R*-gene	Field	332	54.3	304	44.6	304	50.0	170	51.4	137	46.9	3	27	1250		208.3	45.7
	LIFT	0		10	47.6	139	48.9	217	45.4	0	0.0	11	46	377		41.7	46.9
	Total	**332**		**314**		**443**		**387**		**137**		**14**		**1627**			
Number of seedlings selected for LIFT	Field	135		142		13		116		50		3		459		76.5	
	LIFT	0		8		110		123		0		11		252		50.4	
	Total	**135**		**150**		**123**		**239**		**50**		**14**		**711**			
Number of parents in next LIFT generation	Field	9		27		4		0		5		0		45		7.5	
	LIFT	0		1		20		3		0		0		24		4.8	
	Total	**9**		**28**		**24**		**3**		**5**		**0**		**69**			

^*^ % have been calculated only for selected cells; Bold: Totals

The artificial apple scab screening in the greenhouse was primarily used for large progenies to reduce the number of progenies before MAS and “LIFT”. Since larger crosses were mainly done in the field, more seedlings derived from crosses in the field were eliminated during the artificial apple scab screening. Considering both fire blight *R*-genes together, only 11 greenhouse seedlings, compared to 1305 field seedlings, were removed after the artificial scab inoculation. Due to the segregation of the apple scab *R*-gene and the selection of offspring based on the artificial apple scab screening, only about half of the seedlings that resulted from pollination in the field were molecularly tested for the presence or absence of the desired fire blight *R*-gene across generations. Slightly more seeds from the field did not develop or were discarded before they could be tested in MAS compared to the seeds obtained in “LIFT” (39.3% vs 34.9% for *Fb_E* and 26.7% vs 23.9% for *FB_MR5*). In fact, for the large progenies from crosses in the field, before the application of MAS, a stricter selection process was applied to the seedlings in terms of their growth characteristics and health. The number of seedlings carrying either *Fb_E* or *FB_MR5* over all generations ranged from 45.1% to 48% (close to the expected value of 50%), regardless of the source of the seedlings (Table 2).

### Reduction of time between generations

**Fig. 2:**
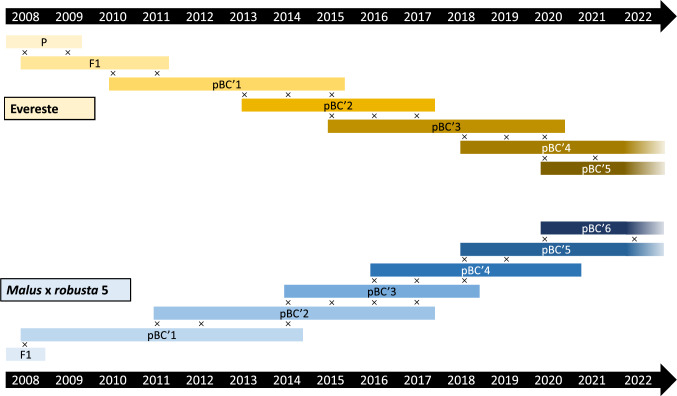
Pseudo-backcross (pBC) schema of the development of the advanced selections of the sixth (pBC’5) and seventh (pBC’6) generations carrying the fire blight *R*-gene *Fb_E* (at the top, starting with ‘Evereste’ as resistance donor) and *FB_MR5* (in the bottom, starting from the F1 of *MR5* as resistance donor), respectively. x: year when crosses were made to generate seedlings of the next generation. See Suppl. Table S1 for the list of all crosses carried out over the years

**Fig. 3:**
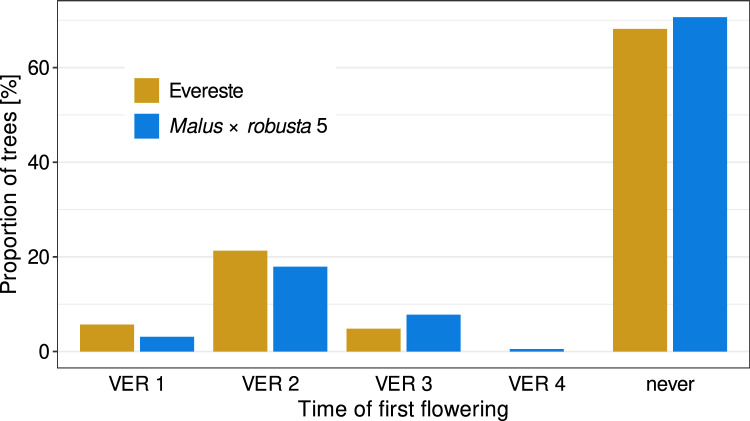
Time to flowering and proportion of seedlings that flowered. VER, number of vernalization period. The data from 354 and 374 seedlings are shown for the introduction series of ‘Evereste’ and *Malus* × *robusta* 5, respectively. Of these 354 and 374 seedlings, 32% and 29%, respectively, flowered at least once within four vernalization periods

Within the analyzed time period (2008–2022), the *Fb_E* introgression series was brought to the sixth generation (pBC’5), and the *FB_MR5* introgression series was brought to the seventh generation (pBC’6) (Fig. 2). On average, the time between generations was reduced to about two years. Across all generations, no more than two vernalization periods were needed in general (Fig. 3) to induce flowering in a sufficient number of seedlings and produce a large enough number of seeds for the next generation (600 to 800 seeds). Once there were sufficient seeds generated for the next generation, the seedlings that did not flower were discarded from “LIFT”.

### Fire blight resistance levels across generations

**Fig. 4:**
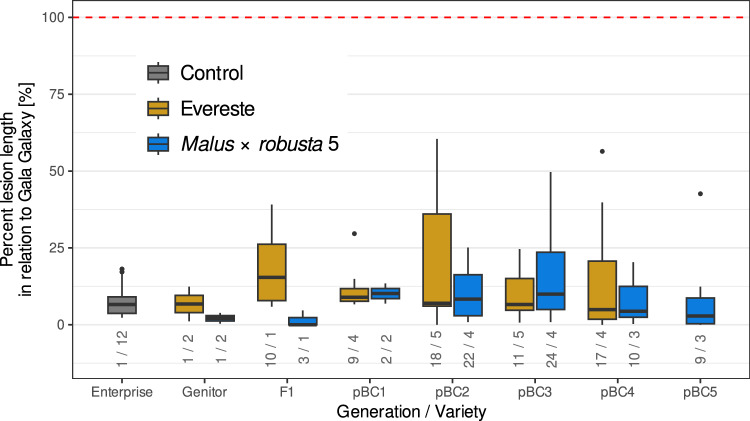
Fire blight resistance level relative to ‘Gala Galaxy’ (set at 100%) 21 days after artificial shoot inoculation. For each generation, the results for multiple tested individuals are shown. The plot displays the mean values per individual and experiment (a few individuals were tested in multiple experiments). The numbers under the boxes indicate the number of tested individuals and the number of trials per generation, respectively. No significant differences between generations were found according to a post hoc Tukey test (*P* < 0.05). ‘Gala Galaxy’ and ‘Enterprise’ were included in each inoculation trial as susceptible and resistant controls, respectively

The level of fire blight resistance of all the seedlings carrying *Fb_E* or *FB_MR5* used as parents was phenotypically assessed (Fig. 4). Although in some generations, individuals with a lower level of fire blight resistance than ‘Evereste’ or MR5 were observed, the average fire blight resistance of all the individuals in a generation did not differ statistically from those of the specific resistance donor (‘Evereste’ or MR5). This was true for all generations and for both fire blight *R*-genes (*Fb_E* and *FB_MR5)*.

### Increase in fruit weight over generations

**Fig. 5:**
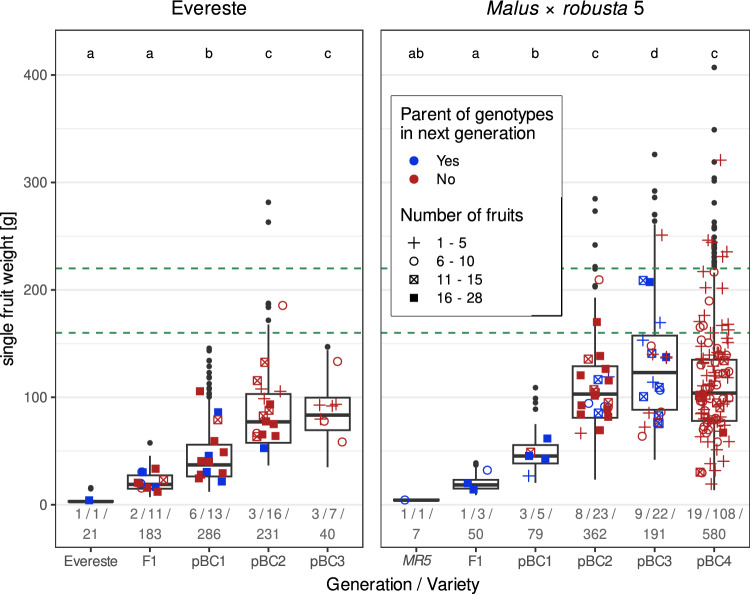
Development of single fruit weight over generations. The boxplots show the weight of single fruits, whereas the symbols show the mean fruit weight per individual. For this, the data from one to three years were aggregated per individual. Individuals were used as parents in the next generations are displayed in blue. The numbers below the boxes indicate the number of crosses per generation, the number of single individuals, and the number of single fruits measured, respectively. Letters indicate significant differences according to a post hoc Tukey test (*P* < 0.05) performed separately for each pseudo-backcross line (*MR5* and ‘Evereste’). The dashed horizontal lines indicate the targeted range of single fruit weight of an optimal dessert apple (160–220 g)

The single fruit weights of “LIFT” individuals that had flowered in “LIFT” and were grafted and planted in the field for conservation were measured on fruits harvested in the field (Suppl. Fig. S1). The median single fruit weight for the individuals carrying the *Fb_E R*-gene increased consistently with each successive generation (Fig. 5). This also applied to the individuals carrying *FB_MR5* up to the pBC’3 generation. Although the median weight of the pBC’4 generation fell back to the level of the pBC’2 generation, several pBC’4 individuals produced fruits heavier than the median of the pBC’3 generation. The first individuals producing fruits larger than 160 g were observed for both *R*-genes starting from the pBC’2 generation.

### Pedigree reconstruction of the parents of ‘Evereste’ and MR5

‘Evereste’ was identified as an offspring of the PRI apple breeding program selection PRI 187–6. The other parent was not identified. Its closest relatives were all exotic *Malus* accessions, particularly the accession *Malus zumi* var. calocarpa (PI 589840 from the USDA Plant Genetic Resources Unit (PGRU) apple collection). No domestic cultivars shared extended haplotypes with the unknown parent of ‘Evereste’. Neither parent was identified for MR5, but ‘Sweet Bough’ (PI 589101 from the USDA PGRU apple collection) was identified as a grandparent. After accounting for the haplotypes MR5 inherited from ‘Sweet Bough’, the other closest relatives of MR5 were mostly *M. baccata* accessions. No domestic cultivars shared extended haplotypes with these haplotypes.

As no *M. domestica* cultivars shared extended haplotypes with the proportions of ‘Evereste’ and MR5 that did not come from their identified ancestors, these proportions were assumed to be of exotic *Malus* origin and were thus assumed to be unadapted. It is therefore estimated that MR5 and ‘Evereste’ are about 75% and 50% unadapted exotic *Malus* origin, respectively.

### Estimation of the proportion of the unadapted exotic ‘Evereste’ and MR5 genome in selected individuals over generations

The exotic *Malus* proportion of ‘Evereste’ and MR5 was estimated in individuals of the pBC’4 and pBC’5 generation, respectively, and in their progenitor carrying the respective *R*-gene (Suppl. Table S7 and S8). In the pBC’5 generation of the *FB_MR5* segregating cross 1817, exotic *Malus* haplotypes comprised, on average, 1.5% (0.5–1.8%) of their genotypic profiles (Suppl. Table S7). Seedling 1817_26 had the smallest exotic *Malus* component remaining, which included the target fire blight *R*-gene *FB_MR5* on chromosome 3. All other individuals carried either only a larger exotic *Malus* haplotype covering the *FB_MR5* locus, with or without a second exotic *Malus* haplotype on chromosome 13.

In the pBC’4 generation of the *Fb_E* segregating cross 1818, higher percentages of exotic *Malus* haplotypes were observed than in 1817. Exotic *Malus* haplotypes comprised, on average, 3.9% (1.2–7.2%) of the genotypic profiles of seedlings from cross 1818 (Suppl. Table S8). Seedling 1818_124 had the lowest amount of exotic genome of ‘Evereste’. This individual showed, besides the exotic *Malus* component closely linked to the target gene *Fb_E* on chromosome 12, small portions of exotic *Malus* from ‘Evereste’, also on chromosomes 7 and 14. Surprisingly, the whole exotic *Malus* chromosome 7 of ‘Evereste’ was maintained up to the fourth generation in cross 1818.

The reduction of the proportion of exotic ‘Evereste’ haplotypes was also studied in individuals carrying *Fb_E* developed with the “early flowering” method. The individuals of the fourth and fifth generations had on average 1.8% and 2.4%, respectively, of their genotypic profiles from the exotic proportion of ‘Evereste’ (Suppl. Table S9). The individuals from the BC3 and BC4 generations with the lowest percentage of remaining exotic ‘Evereste’ haplotypes were BC_3_2014_17 and BC_3_2014_36 (both 1.1%) and BC_4_79 (1.3%), respectively. In these BC3 individuals, there were only two exotic ‘Evereste’ haplotypes remaining — one covering 3.6% of chromosome 12 containing the *Fb_E R*-gene and another covering about 25% of chromosome 9 — whereas the exotic haplotype on chromosome 9 was absent in BC_4_79. However, this individual was still carrying about one third of chromosome 12 containing the *Fb_E R*-gene.

## Discussion

### The “LIFT” method

The “LIFT” method successfully reduced the time between generations. The majority of the seedlings that flowered produced flowers after the second vernalization period, i.e., about two years after sowing (Fig. 3). This corresponds to about half of the time that a cross-bred progeny normally requires to produce its first flowers in the open field in a classical breeding scheme. Moreover, it is similar to the reduction of time reached by Volz et al. (2009), who applied a much more sophisticated protocol. However, only about 20% of the trees flowered after the second vernalization period (Fig. 3). Thus, a relatively large number of seeds have to be produced in each generation. At different steps of the protocol, a loss of material or efficacy was observed. Compared to “LIFT” grown trees, trees in the field typically produced more flowers and therefore more fruits and seeds (Table 2 and 3). Therefore, pollen collected from the flowers of the most advanced generation of seedlings in “LIFT” can be used very efficiently to produce larger offspring populations in the field. One possible reason for the lower number of flowers on seedlings in “LIFT” could be the generally high stress levels of the plants due to the applied cultivation method (e.g., relatively small pot, dense stand, and competition for light) and the lack of measures to promote fertility, such as those used in modern fruit tree cultivation (e.g., grafting on different rootstocks, and pruning technique) (Martin 2020). However, it was observed that over the generations, slightly more seeds per fruit were produced from “LIFT” trees (Tables 2 and 3). This could be due to the slightly better quality of the pollen from the field or the generally good conditions for fertilization in the greenhouse.

Many of the cultural management practices included in the protocol (sowing date, duration of vegetative growth and vernalization, artificial light setting, fertigation, timing, and concentration of Regalis® and Ethephon application) have not yet been fully optimized and exploited, hence the name “low input fast-track” (LIFT) method. Often, the time and effort required for “LIFT” had to be adapted to the prevailing work in conventional apple breeding and the infrastructural possibilities. Additional cultural management, such as those described by Volz et al. (2009) or the use of specific light spectra, as described for *Solanum lycopersicum* (Giliberto et al. 2005) and *Lactuca sativa* (Johkan et al. 2010), were not tested.

In both introgression series, more seedlings originating from the field were eliminated during the artificial apple scab screening than from “LIFT” (Tables 2 and 3), for three reasons. First, there was only space for a limited number of seedlings in the “LIFT” system; therefore, a strict selection was necessary. Second, a sufficiently large number of seedlings per cross, which allows for the selection of two traits (scab and fire blight resistance), was generally only achieved with the crosses made in the field. Third, there was a constraint in terms of when the seedlings could be tested in the artificial apple scab screening, which takes place once a year in spring as part of the conventional breeding program. Due to the relatively low number of seedlings produced in “LIFT”, it was not worth inoculating them in an ad hoc artificial apple scab screening to remove the few that were scab susceptible. *R*-genes for apple scab were also identified during MAS and used for selection when it was assumed that a sufficiently large number of seedlings carrying the fire blight *R*-gene (*Fb_E* or *FB_MR5*) were available to start the next generation in the “LIFT” cycle.

Considering all these possible pitfalls and aspects and the fact that about 50% of the seedlings are discarded based on MAS because they do not carry the fire blight *R*-gene, we recommend producing 600–800 seeds per generation from different cross combinations to obtain between 30 and 40 seedlings that will potentially start to flower after the second vernalization cycle in “LIFT”.

### Non-eroding fire blight resistance and increase in fruit weight over generations

Among the 65 *Fb_E* individuals tested for fire blight resistance, there were18 with median fire blight resistance scores higher (30% lesion length) than the expected value for *Fb_E* (i.e., very close to zero) (Fig. 4). Interestingly, the progeny plants that directly descended from these individuals fully regained the level of high fire blight resistance expected for *Fb_E*. This behavior indicates that a second gene may be required to reach the high fire blight resistance of *Fb_E*, and that its favorable alleles occur relatively frequently in the individuals examined in our experiment. Parravicini et al. (2011) identified two candidate resistance genes, *MdE-EaN* and *MdE-EaK7*, with similarities to *Prf* and *Pto* in the *Fb_E* region. Parravicini et al. (2011) speculated that the genes may work together to confer resistance, as shown in tomato. This hypothesis needs to be tested in further studies. In addition, in the descendant generations of MR5, 8 out of 70 individuals showed a susceptibility of more than 30% lesion length compared to that of MR5 (i.e., very close to zero) and were thus more susceptible than expected (Fig. 4). Although *FB_MR5* induces a very high level of fire blight resistance, this *R*-gene should be used only in combination with at least one other fire blight resistance gene, as the resistance of MR5 (*FB_MR5*) is already known to be overcome. A single point mutation in the *avrRpt2*_*EA*_ gene of *E. amylovora* was sufficient to break the resistance (Vogt et al. 2013).

Single fruit weight increased rapidly and consistently on average for both *R*-genes up to the pBC’3 generation, with some individuals of the third generation (pBC’2) producing fruits in the target range (160–220 g) (Fig. 5). For both introgression series, a reduction in the increase of single fruit weight was observed from the pBC’2 generation onwards, and the mean single fruit weight of the pBC’4 generation of *FB_MR5* was significantly lower than that of the pBC’3 generation. Nevertheless, an acceptable proportion of individuals of this generation (pBC’4 *FB_MR5*) produced fruits in the target range. A much larger number of single fruits from many more progenies were measured for pBC’4 compared to pBC’3 of *FB_MR5* (Fig. 5). Some of the greater variability in single fruit weight can, therefore, be attributed to the larger sample size. Among the individuals with a single fruit weight within the target range from the pBC’4 of ‘Evereste’ and pBC’5 of MR5, those with the best performance for other characteristics will be identified in the future and included as elite parents in the regular breeding program.

### Pedigree reconstruction of ‘Evereste’ and MR5 and the reduction of their exotic genome using the “LIFT” protocol

Pedigree reconstruction of MR5 and ‘Evereste’ enabled more precise and useful tracking of the unadapted exotic *Malus* portions of their genomes through the introgression series. The pedigree of the species *Malus* × *robusta* was previously unknown but expected to be fully exotic, considering it was recorded as a hybrid between *M. baccata* and *M. prunifolia* (Fialla 1994). However, in our study, the dessert variety ‘Sweet Bough’ was identified as the grandparent of MR5. The pedigree of ‘Evereste’ was recorded to be a cross between PRI 187–11 and an unknown parent from which *Fb_E* derives. PRI 187–11 was unavailable for genotyping, but the selection PRI 187–6 matched as the parent instead. PRI 187–6 is an advanced breeding selection derived from quality apple cultivars, i.e., Rome Beauty, Jonathan, and Red Delicious. We considered the portion of ‘Evereste’s genome inherited from PRI 187–6 as adapted, although it contains some haplotypes from *M. floribunda* 821, which was in its pedigree as a source for the *Rvi6* (*Vf*) apple scab *R*-gene (Crosby et al. 1992). Haplotypes from the other portions of MR5 and ‘Evereste’, 75% and 50%, respectively, did not have any clear *M. domestica* relatives and were most similar to accessions of *M. baccata* and thus were considered to be of unadapted exotic *Malus* origin. By labeling those areas of the genome as unadapted, it was possible to track and select against them. In this study, this selection was done at the end of the introgression series, but this could be accomplished during the introgression process to more efficiently select for or against individuals with a higher or lower proportion of unadapted haplotypes, respectively, as first described by Hospital and Charcosset (1997).

The reduction of the part of the unadapted exotic genome of the resistance donors progressed as expected over the generations in the case of the introduction series of *Fb_E*. The analyzed “LIFT” F1 of ‘Evereste’ comprised 29.7% of exotic *Malus* haplotypes, while the F1 developed with the “early flowering” approach had 23.1% and therefore very close to the expected 25%. This also applies to “LIFT” pBC’4 from ‘Evereste’, which had an average of 3.9% exotic *Malus* haplotypes, and those developed by “early flowering”, which had 2.4%. Both were close to the expected 3.1%.

In the case of the introduction series of *FB_MR5*, the only F1 analyzed was 6.1% above the expected 37.5%, but the 37 seedlings of the pBC’5 generation had, on average, 1.5% of their genotypic profiles comprised of unadapted exotic *Malus* haplotypes, which was below the expected 2.3%. In 21 out of the 37 pBC’5 seedlings, the only unadapted exotic haplotype remaining contained *FB_MR5*. No such seedling was available among the most advanced *Fb_E* generation, i.e., pBC’4. However, four *Fb_E* pBC’4 seedlings carrying a single fragment of the unadapted exotic genome, in addition to the unadapted exotic genome associated with the *Fb_E* locus, were identified. If one of these would flower sufficiently fast, statistically, about one quarter of the progenies of the next generation will carry only the unadapted exotic *Malus* haplotype containing *Fb_E*. These seedlings would be the first individuals carrying only the *R*-gene *Fb_E* and could be introduced into the regular breeding program. Alternatively, these seedlings could be kept for an additional round in “LIFT” and crossed with similar seedlings, i.e., individuals carrying only unadapted exotic genome associated with the *R*-gene *FB_MR5*.

Surprisingly, during the application of “LIFT” for *Fb_E*, the whole unadapted exotic chromosome 7 of ‘Evereste’ was inherited up to the seedlings of the pBC’3 generation (e.g., 1609_24). Furthermore, a large part of chromosome 17 was inherited intact over five generations (Suppl. Table S8). Similarly, in the “LIFT” for *FB_MR5*, about half of the exotic chromosome 10 was conserved over three generations, and additional conserved fragments over the generations were located on different chromosomes. We anticipate that these results are probably due to random effects. However, it could also be due to the presence of genes that may play a role in inducing precocious flowering, the most important selection criteria applied in “LIFT”.

In this study, the removal of non-adapted exotic *Malus* haplotypes in ‘LIFT’ individuals was monitored in individuals of the fifth (‘Evereste’) and sixth (MR5) generations. Le Roux et al. (2012) estimated the percentage of unadapted genome in the seedlings of the second generation (pBC’1) while applying the “early flowering” approach based on the T1190 line. They found two seedlings, BC1_16 and BC1_19, with 13.8% and 14.4% of unadapted exotic *Malus* haplotypes of ‘Evereste’ remaining, well below the average of 25% expected for the pBC’1 generation. Note that in Le Roux et al.’s (2012) study, the entire genome of ‘Evereste’ was considered unadapted, as its pedigree was not known/confirmed at the time. For this reason, both seedlings were prioritized in subsequent crosses. The same approach, i.e., the addition of a background selection at each generation to identify the seedlings with the lowest percentage of unadapted exotic genome, could also be combined with “LIFT”. The application of background selection in early generations could potentially help to reduce the overall number of pseudo-backcrosses. However, some aspects need to be considered. Compared to “early flowering” seedlings, only about 20% of “LIFT” seedlings will flower sufficiently early to save time between generations. Therefore, if a background selection is applied at an early stage, a sufficiently large number of “LIFT” seedlings have to be screened and kept. Moreover, the costs of the background selection of a large number of plants may be too high to economically justify application compared to keeping more plants with an unknown genetic background in the greenhouse. To reduce background selection costs, instead of using a large number of SNP markers spanning the whole genome, as applied in this study, a further reduction of genome representation could be achieved by using a selected set of KASP™ markers. Alternatively, if the identification of individuals more likely to not have linkage drag were to be prioritized, a set of markers spanning only the chromosome containing the gene of interest could be used, such as the six SSRs used by Schlathölter et al. (2018).

## Conclusion

Since the introduction of the “LIFT” method in 2008, six generations of introgression cycles have been achieved in 15 years. This results in a halving of the generation time compared to normal cross breeding in the field. Thus, the “LIFT” poses a powerful protocol for reducing the time between generations and it can be used to remove the unadapted, exotic *Malus* genomes introduced into a breeding program together with resistance found in these materials. MR5 pBC’5 seedlings carrying only exotic *Malus* haplotypes flanking *FB_MR5* have been identified those carrying only *Fb_E* are expected to become available in the next generation, i.e., pBC’5. The fruit weight of these seedlings has now reached levels that should ensure that a large proportion of the next generation will produce fruit of sufficient size to meet market requirements. Stacking the *Fb_E* and *FB_MR5 R*-genes carried by the advanced selections developed in this work will pave the way for the development of urgently needed new cultivars with durable fire blight resistance.

## Supplementary Information

Below is the link to the electronic supplementary material.

Supplementary file1 (XLSX 20 KB)

Supplementary file2 (XLSX 18 KB)

Supplementary file3 (XLSX 16 KB)

Supplementary file4 (XLSX 4034 KB)

Supplementary file5 (XLSX 14 KB)

Supplementary file6 (XLSX 13 KB)

Supplementary file7 (XLSX 20 KB)

Supplementary file8 (XLSX 20 KB)

Supplementary file9 (XLSX 19 KB)

Supplementary file10 (PDF 371 KB)

## Data Availability

All data supporting the findings of this study are available within the paper and its supplementary information.

## Abbreviations

*Fb_E*: Fire-blight R-gene of ‘Evereste’
*FB_MR5*: Fire-blight R-gene of *Malus* × *robusta* 5
LIFT: Low input fast-track
MAS: Marker-assisted selection
MR5: *Malus × robusta* 5
pBC(s): Pseudo-backcross(es)
PRI: Purdue, Rutgers, Illinois
SNP: Single nucleotide polymorphism
SSR: Simple-sequence repeat
