# Supplementary material for: Low input fast-track (LIFT): an approach for fast introgression and stacking of (R-)genes into advanced apple selections
Source: Planta. 2025 Sep 2;262(4):93. doi: 10.1007/s00425-025-04780-4 (PMC12405375; doi:10.1007/s00425-025-04780-4)
Supplement: Supplementary file 10 — Supplementary file10 (PDF 371 KB) [file 425_2025_4780_MOESM10_ESM.pdf]

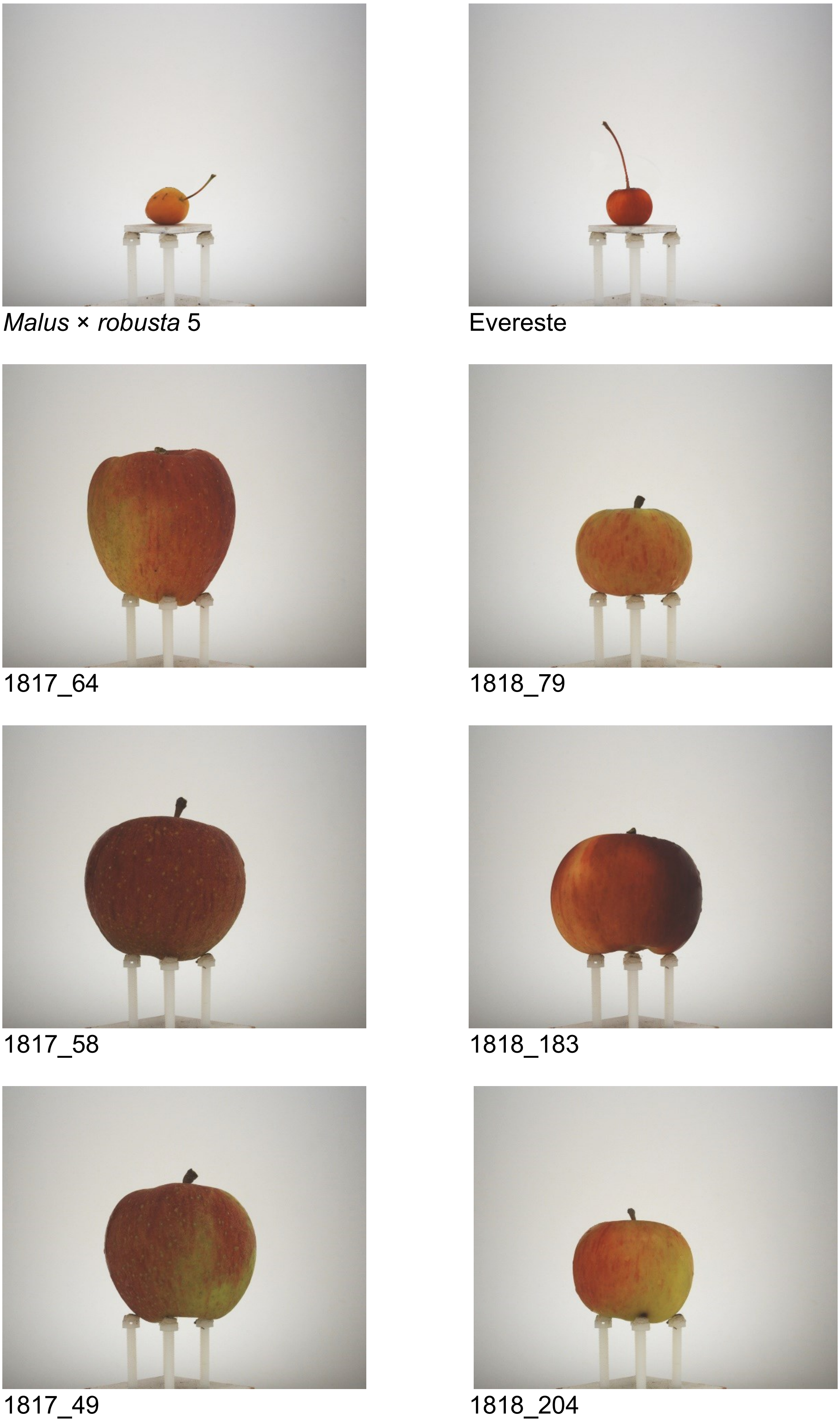

Suppl. Fig. S1 Representative photos of fruits from *Malus × robusta* 5, 'Evereste', along with three examples of offspring from the fifth pseudo backcross generation (pBC'5) from *Malus × robusta* 5 (cross combination 1817) or pBC'4 from 'Evereste' (cross combination 1818) taken with the FruitPhenoBox (Kirchgessner et al. 2024) of fruits harvested from trees grown in the field in 2023 or 2024 at Agroscope in Waedenswil, Switzerland.
